# The Identification of a Target Gene of the Transcription Factor KojR and Elucidation of Its Role in Carbon Metabolism for Kojic Acid Biosynthesis in *Aspergillus oryzae*

**DOI:** 10.3390/jof10020113

**Published:** 2024-01-30

**Authors:** Tomoka Mizutani, Hiroya Oka, Riko Goto, Ryoga Tsurigami, Jun-ichi Maruyama, Motoyuki Shimizu, Masashi Kato, Hideo Nakano, Takaaki Kojima

**Affiliations:** 1 Department of Applied Biosciences, Graduate School of Bioagricultural Sciences, Nagoya University, Furo, Chikusa, Nagoya 464-8601, Aichi, Japanoka.hiroya.n1@f.mail.nagoya-u.ac.jp (H.O.); hnakano@agr.nagoya-u.ac.jp (H.N.); 2 Department of Agrobiological Resources, Faculty of Agriculture, Meijo University, Shiogamaguchi, Tempaku, Nagoya 468-8502, Aichi, Japan; 3 Department of Applied Biological Chemistry, Faculty of Agriculture, Meijo University, Shiogamaguchi, Tempaku, Nagoya 468-8502, Aichi, Japanmoshimi@meijo-u.ac.jp (M.S.); mkato@meijo-u.ac.jp (M.K.); 4 Department of Biotechnology, The University of Tokyo, 1-1-1 Yayoi, Bunkyo, Tokyo 113-8657, Japan; amarujun@g.ecc.u-tokyo.ac.jp; 5 Collaborative Research Institute for Innovative Microbiology, The University of Tokyo, 1-1-1 Yayoi, Bunkyo, Tokyo 113-8657, Japan

**Keywords:** transcriptome, transcription factor, *Aspergillus oryzae*, kojic acid (KA), bioinformatics

## Abstract

DNA-binding transcription factors are broadly characterized as proteins that bind to specific sequences within genomic DNA and modulate the expression of downstream genes. This study focused on KojR, a transcription factor involved in the metabolism of kojic acid, which is an organic acid synthesized in *Aspergillus oryzae* and is known for its tyrosinase-inhibitory properties. However, the regulatory mechanism underlying KojR-mediated kojic acid synthesis remains unclear. Hence, we aimed to obtain a comprehensive identification of KojR-associated genes using genomic systematic evolution of ligands by exponential enrichment with high-throughput DNA sequencing (gSELEX-Seq) and RNA-Seq. During the genome-wide exploration of KojR-binding sites via gSELEX-Seq and identification of KojR-dependent differentially expressed genes (DEGs) using RNA-Seq, we confirmed that KojR preferentially binds to 5′-CGGCTAATGCGG-3′, and KojR directly regulates *kojT*, as was previously reported. We also observed that *kojA* expression, which may be controlled by KojR, was significantly reduced in a *ΔkojR* strain. Notably, no binding of KojR to the *kojA* promoter region was detected. Furthermore, certain KojR-dependent DEGs identified in the present study were associated with enzymes implicated in the carbon metabolic pathway of *A. oryzae*. This strongly indicates that KojR plays a central role in carbon metabolism in *A. oryzae*.

## 1. Introduction

Kojic acid (KA) is highly valuable in several industries. For example, in the cosmetics industry, it is popular for use in skin lightening applications owing to its ability to inhibit tyrosinase and reduce melanin production [1]. Additionally, the antibacterial, antifungal, and anti-inflammatory properties of KA and its derivatives increase its potential for use in the pharmaceutical industry [2].

In Japan, *Aspergillus oryzae* has been historically used in the brewing and food industries, produces KA as a secondary metabolite [3], and can be used in fermentative KA production. However, the specific pathway for KA synthesis in *A. oryzae* remains unclear, despite the suggestion of various possible pathways. In fact, a consensus is yet to be reached even a century after the initial observation was made [3]. An interesting review on the fungal biosynthesis of KA was published very recently, and the article presented the metabolic pathways of KA [4]; however, the genes pertaining to each metabolic reaction are yet to be identified.

*A. oryzae* houses *kojR* (AO090113000137), which encodes a Zn(II)_2_Cys_6_ transcriptional activator that is integral to KA biosynthesis [5,6]. *kojR* deletion significantly downregulates KA synthesis [6,7]. *kojR* is located between *kojA* (AO090113000136) and *kojT* (AO090113000138) and plays a pivotal role in regulating their expression [5,6,7]. Chang et al. identified an 11-bp palindromic sequence within the *kojA* and *kojT* promoter regions in 23 *Aspergillus* species including *A. oryzae* and *A. flavus* [8]. They prepared *A. flavus* mutants using CRISPR/Cas9 by deleting the binding regions inferred from the palindromic sequence in the *kojA* and *kojT* promoters and showed that several of these mutants lost their ability to synthesize KA [8]. These results have suggested that KojR directly regulates *kojA* and *kojT* expression in *A. flavus*.

Additionally, KojR is involved in gliotoxin biosynthesis in *A. fumigatus* and protects against gliotoxin in *A. fumigatus*, *A. nidulans*, and *A. oryzae* [9], implying that the regulatory mechanism of KojR is not confined to the group of genes associated with KA biosynthesis. However, the KojR binding sites in the genome and the genes whose expression is directly regulated by KojR have not been comprehensively investigated.

We have previously used the genomic systematic evolution of ligands by exponential enrichment with high-throughput DNA sequencing (gSELEX-Seq) technique and a differential expression analysis method, such as DNA microarray analysis or RNA-Seq, and established a system for the comprehensive analysis of transcriptional regulatory networks. This approach has enabled us to successfully identify the direct target genes of a specific transcription factor (TF) [10,11]. Furthermore, based on the information obtained using the analysis system regarding the binding sites of an *A. oryzae* TF, AoXlnR, we have shown that the type and number of binding sites correlates with the level of differential expression and that the AoXlnR-dependent differential expression of downstream genes can be predicted based on the parameters of the DNA structure in the vicinity of the binding sites [11,12].

In this study, we aimed to elucidate the mechanism of KojR-mediated KA synthesis in *A. oryzae* through the genome-wide investigation of KojR binding sites using gSELEX-Seq and identification of KojR-dependent differential expression genes (DEGs) via RNA-Seq with a *ΔkojR* strain. Our findings indicate that *kojT*, which encodes a putative transporter in *A. oryzae*, is directly regulated by KojR. We further examined the C-source metabolic pathway in *A. oryzae* and analyzed its connection to the DEGs regulated by KojR.

## 2. Materials and Methods

### 2.1. A. oryzae Strains, Growth Conditions, and Transformation

Wild-type *A. oryzae* strain RIB40 [13] was used in this study. The transformation of *A. oryzae* strains was performed as follows: The *A. oryzae* RIB40 strain was seeded and incubated overnight at 37 °C. The mycelia were collected with a nylon mesh filter (110 mesh), washed with 0.8 M NaCl, and incubated with 20 mL × 2 protoplastogenic solution [0.3% Yatalase (Takara, Kusatsu, Shiga, Japan), 0.03% Lysing enzyme from *Trichoderma harzianum* (Sigma-Aldrich, St. Louis, MO, USA), 0.8 M NaCl, and 10 mM sodium phosphate buffer (pH 5.8)] with shaking at 30 °C for 2 h. The protoplastidized solution was consecutively filtered through 110 and 305 nylon mesh filters and centrifuged (1500 rpm, 5 min, at RT) using a swinging rotor to remove the supernatant. The obtained pellet was washed with 0.8 M NaCl and centrifuged using a swinging rotor (1500 rpm, 5 min, at RT) to remove the supernatant. The washing step was repeated twice. Next, 10 mL of Sol I (0.8 M NaCl and 10 mM CaCl_2_) was gently added, and the mixture was centrifuged using a swing rotor (1500 rpm, 5 min, at RT). Most of the supernatant was removed but approximately 300–500 μL was retained, and the pellet was resuspended in the remaining supernatant. The DNA of interest for transformation (approximately 10 µg) and 12.5 µL of Sol II (0.25 g/mL PEG6000, 50 mM CaCl_2_, and 10 mM Tris-HCl (pH 7.5)) were added to 50 µL of the protoplast suspension, which was gently suspended and placed on ice for 20 min. An additional 500 µL of Sol II was added, gently suspended, and allowed to stand on ice for 5 min. Then, 900 µL of Sol I was added and gently suspended. The suspensions were inoculated onto plates with regeneration media (70 mM NaNO_3_, 11 mM KH_2_PO_4_, 7 mM KCl, 22% (*w*/*v*) sorbitol, 2% (*w*/*v*) D-glucose, 0.15% (*v*/*v*) trace element solution, 2 mM MgSO_4_, and 0.01% pyrithiamine (pH 6.5)) and incubated at 37 °C for several days.

### 2.2. The Construction of a KojR FL/BD Expression Vector

The gene encoding the KojR constructs was sourced from gBlock Gene Fragments (Integrated DNA Technologies, Coralville, IA, USA). Using this construct as the template, fragments for kojR or kojR-BD were amplified using HO-kojR-F and HO-kojR-R, or using HO-kojR-F, and HO-kojR-BD-R (Supplementary Table S1). These fragments were then cloned through the SLiCE method [14] into a vector fragment amplified with PMAL-IFC-F1 and PMAL-IFC-R1 (Supplementary Table S1) using pMalc2AmyR_1–411_ [15] as the template (pMalc2KojR or pMalc2KojR-BD).

A plasmid construct for the cell-free expression of maltose binding protein (MBP)-fused full-length KojR was prepared using the following procedure. The full-length KojR gene fragment was amplified using kojR-F and kojRFull-R (Supplementary Table S1) with pMalc2KojR as the template. The amplicon was cloned using the NEBuilder HiFi DNA Assembly Master Mix (New England BioLabs, Ipswich, MA, USA) into a vector fragment amplified with gibson_Full-F and gibson_kojR-R (Supplementary Table S1) using pRSETb-MBP-DrDsxF (Kojima, T., unpublished) as the template (pRSETb-MBP-KojR-FL).

A plasmid construct for the cell-free expression of MBP-fused KojR_1–70_ (pRSETb-MBP-KojR-BD) was prepared using the procedure described above with the following exceptions: the N-terminal region of the KojR gene fragment was amplified using kojR-F and kojRBD-R (Supplementary Table S1) with pMalc2KojR-BD as the template. The vector fragment was amplified with gibson_BD-F and gibson_KojR-R primers (Supplementary Table S1) using pRSETb-MBP-DrDsxF as the template.

### 2.3. The Expression of MBP-Fused KojR Using a Cell-Free Protein Synthesis System

Template DNA for the cell-free protein expression of MBP-fused full-length KojR or MBP-fused KojR_1–70_ was prepared using PCR with F1-primer and R1-primer (Supplementary Table S1); pRSETb-MBP-KojR-FL or pRSETb-MBP-KojR-BD was used as the template, respectively. Cell-free protein synthesis was performed using the PUREfrex 2.1 kit (GeneFrontier, Kashiwa, Chiba, Japan) according to the manufacturer’s instructions. Briefly, cysteine, GSH, Solution I, Solution II, and Solution III from the kit, template DNA for the synthesis of MBP-fused full-length KojR or MBP-fused KojR_1–70_, and ZnCl_2_ (final conc. 2 mM) were mixed to obtain a reaction mixture (10 µL). The mixture was incubated at 37 °C for 2 h and centrifuged at 15,000 rpm for 5 min at RT to separate the soluble and insoluble fractions. The soluble fraction was used for the binding reaction in gSELEX (described in the next section).

SDS-PAGE was performed to confirm the expressed proteins using a cell-free protein synthesis system, and 0.1 μL of fluorescently labeled lysine tRNA (FluoroTect GreenLys tRNA, Promega, Tokyo, Japan) was added to the cell-free protein synthesis reaction mixture to visualize the expressed protein. The insoluble fraction recovered after the reaction was suspended in 10 μL of 1 × PBS and applied to the analytical gel. A scanner capable of detecting green fluorescence was used to visualize the expressed proteins. The Protein Molecular Weight Marker (Takara) was fluorescently labeled using the Fluorescein Labeling Kit-NH_2_ (Dojindo Laboratory, Kumamoto, Japan) and used as the molecular weight marker.

### 2.4. The In Vitro Selection of KojR-Bound DNA Fragments from an A. oryzae Genomic Library Using gSELEX-Seq

The *A. oryzae* genomic library was prepared according to a previously reported procedure [11]. gSELEX was performed using the *A. oryzae* genomic library. First, 9 µL of cell-free expressed KojR FL or KojR BD solution and 1 µL of 5 ng/µL *A. oryzae* genomic library solution were mixed gently by rotation for 30 min at RT. Next, 5 μL of amylose resin (New England BioLabs) was washed with 50 μL of MBP *w*/*o* EDTA buffer (200 mM NaCl, 20 mM Tris–HCl, and 10 mM 2-mercaptoethanol (pH 7.5)). The resin was suspended in a 1.5-mL tube in 90 μL of fresh MBP *w*/*o* EDTA buffer and mixed with the MBP-KojR FL/BD-binding reaction mixture. The suspension was mixed overnight using a rotator at 4 °C, following which the resin was recovered through centrifuging at 300× *g* for 2 min at 4 °C. The resin was washed with 50 μL of MBP *w*/*o* EDTA buffer to remove the free DNA fragments. After removing as much of the supernatant as was possible, the resin was suspended in 10 μL of MBP *w*/*o* EDTA elution buffer (200 mM NaCl, 20 mM Tris–HCl, 10 mM 2-mercaptoethanol, and 20 mM maltose (pH 7.5)). The suspension was mixed using a rotator for 15 min at 4 °C, and the supernatant was recovered after centrifugation at 300× *g* for 2 min at 4 °C. The selected DNA fragments were amplified using a PCR with Nextera-Read1 and Nextera-Read2 (Supplementary Table S1). The following program was used: preheating at 94 °C for 3 min; 17 cycles of 94 °C for 15 s, 68 °C for 10 s, and 72 °C for 20 s; and a final extension at 72 °C for 5 min. After purification, the DNA pool was used for the second round of selection, in which the selected DNA pool was prepared using the same procedure as that used in the first round with the following exception: 5 ng of the DNA pool (selected in the first round) and 1 mU of poly(deoxyinosinic-deoxycytidylic) acid sodium salt (polydIdC) (Sigma-Aldrich) were added to the binding reaction mixture containing the cell-free KojR. In the third round, 5 ng (KojR FL) or 7.5 ng (KojR BD) of the DNA pool selected in the second round was used with 1 mU of polydIdC in the binding reaction. The concentration of each purified DNA pool was assessed using the Quant-iT dsDNA Broad-Range Assay Kit (Thermo Fisher Scientific, Waltham, MA, USA) according to the manufacturer’s instructions.

### 2.5. DNA Sequencing and Data Analysis in gSELEX-Seq

Sequencing libraries using each DNA pool obtained using gSELEX were prepared with the NEBNext Ultra II DNA Library Prep Kit for Illumina and NEBNext Multiplex Oligos for Illumina (New England BioLabs). The libraries were applied to Illumina NextSeq550 to obtain sequence data in 81 b read length in paired-end mode. It should be noted that the DNA pool from the unselected *A. oryzae* genomic library was also sequenced for use as a control tag in detecting the peaks of the KojR-binding site. All sequencing data will be made available through the DNA Databank of Japan (DDBJ; Bioproject accession number: PRJDB16474).

The genome-wide analysis of KojR binding sites using sequencing data obtained using NextSeq 550 was performed according to the following procedure: (1) The 5′ adapter was stripped from the reads using Cutadapt (ver. 4.3) [16] with the following parameters: -g AGATGTGTATAAGAGACAG -g CTGTCTCTTATACACATCT, followed by the removal of the unpaired reads using Trimmomatic (ver. 0.39) [17]. (2) The trimmed paired-end reads were mapped using Bowtie2 (ver. 2.2.3) [18] into an *A. oryzae* genome file (aor0-5.fna) obtained from the Comprehensive *Aspergillus oryzae* Genome Database (CAoGD) (https://nribf21.nrib.go.jp/CAoGD/ (accessed on 18 May 2022)) using default settings. The generated SAM files were converted into BAM files using SAMtools (ver. 0.1.18) [19]. (3) HOMER (v4.11.1) findPeaks (http://homer.ucsd.edu/homer/ngs/peaks.html (accessed on 11 April 2023)) was used to identify KojR FL or KojR BD binding sites with the following parameters: -i control tag directory -LP 0.01 -P 0.01. This was followed by the preparation of a bed file based on the position values of detected peaks. Additionally, the motif analysis of KojR FL- or KojR BD-bound sequences was performed using Homer findMotifsGenome.pl (http://homer.ucsd.edu/homer/ngs/peakMotifs.html (accessed on 11 April 2023)) based on the acquired peak regions. (4) The obtained bed files were converted into FASTA files using the aor0-5.fna and BEDTools fastaFromBed (ver. v2.17.0) [20]. (5) The promoters possibly regulated by KojR FL or KojR BD were annotated as follows: the sequences of the peak regions obtained from the third selection round were annotated using the *A. oryzae* upstream 1000 dataset [11] and BLAST+ (ver. 2.13.0+) (https://blast.ncbi.nlm.nih.gov/doc/blast-help/downloadblastdata.html (accessed on 11 November 2022)) with the following parameters: blastn -evalue 0.1 -outfmt 6.

### 2.6. The Construction of an A. oryzae ΔkojR Strain Using CRISPR/Cas9

A mutation was introduced into the KojR-binding domain-coding region of the *A. oryzae* RIB40 genome using the CRISPR/Cas9 system reported in a previous study [21]. Linear DNA with the *wA* target site of ppAsAC9gwA [21] replaced with a 21 bp *kojR* target site was prepared using PCR with ppAsAC9gwA as the template along with ppAsAC9gwA_F7 and ppAsAC9gwA_R7 (Supplementary Table S1). The linear fragment was re-circled using the SLiCE method [14] and introduced into *Escherichia coli* DH5α for selection to obtain the plasmid ppAsAC9g_kojR_BD for the preparation of the *ΔkojR* strain. ppAsAC9g_kojR_BD was transformed into the *A. oryzae* RIB40 strain to introduce mutations into the *kojR* target site in the genome. The mycelia of the transformants on the regeneration medium plate were collected, suspended in 50 µL of sterilized water, and incubated at 95 °C for 15 min. The suspension was used as the template for the amplification of the target mutation site in *kojR* using PCR using the kojR_CP_F and kojR_CP_R primers (Supplementary Table S1). The amplified products were subjected to DNA sequencing analysis using the kojR_seq primer (Supplementary Table S1) for the investigation of mutation and gene deletion/integration. The loss of the KojR function of the *ΔkojR* strain was confirmed using the non-pigmentation of the culture medium after 3 days of incubation in the KA medium containing FeCl_3_. Note that FeCl_3_-containing medium turns red due to the chelating of KA.

### 2.7. The Identifications of DEGs Associated with KojR Expression Using RNA-Seq

The spores of *A. oryzae* RIB40 or the *ΔkojR* strain were inoculated (5–6 × 10^6^) into 50 mL of KA production medium (10% (*w*/*v*) D-glucose, 0.25% (*w*/*v*) yeast extract, 5 mM K_2_HPO_4_, and 4 mM MgSO_4_ (pH 6.0)) [6] with 1 mM FeCl_3_ and incubated with shaking for 3 days at 37 °C. The cultured mycelia were collected, frozen at −80 °C, divided into approximately 0.1 g samples that were added to each 2-mL screw cap tube, and disrupted using Multi-beads shocker (Yasui Kikai, Osaka, Japan) (2500 rpm, 10 s; off time 3 s × 3 cycles). Total RNA was extracted from each disrupted mycelium using a PureLink RNA Mini Kit (Thermo Fisher Scientific) according to the manufacturer’s instructions.

cDNA fragments of six samples (3 × RIB40 and 3 × *ΔkojR*) were subjected to sequencing analysis using the MinION nanopore sequencer (Oxford Nanopore Technologies, Oxford, UK). Each cDNA library was prepared using the cDNA-PCR Sequencing Kit (SQK-PCS109) and PCR Barcoding Kit (SQK-PBK004) (Oxford Nanopore Technologies) according to the manufacturer’s protocol. However, one of the RIB40-derived samples was not used in differential expression analyses because it did not yield a sufficient amount of count data. All sequencing data will be made available through the DNA Databank of Japan (DDBJ; Bioproject accession number: PRJDB16475).

The sequenced data obtained using MinION was subjected to differential expression analysis as follows: (1) Polythymine and polyadenine sequences at the end of the reads were sequentially removed using Cutadapt (ver. 4.3) [16] with the following parameters: -g TTTTTTTT -n 20 -O 10; -a AAAAAAAAAA -n 10 -O 10. (2) Mapping was performed using minimap2 (ver. 2.17-r954-dirty) [22] with the following parameters: -ax splice -m 20 using the *A. oryzae* genome file (aor0-5.fna). The generated SAM files were converted into BAM files using SAMtools (ver. 0.1.18) [19]. (3) The counts of reads mapped to the coding sequences (CDS) of genes in the *A. oryzae* genome were performed using FeatureCounts (ver. 1.5.2) [23] with the following parameters: --readExtension5 200 --readExtension3 200 using an *A. oryzae* GTF file (aor0-5.gtf) obtained using CAoGD. The --readExtension5 and --readExtension3 arguments were specified to account for the lower counts due to the degradation of RNA applied to sequencing. False discovery rate (FDR) was calculated using the χ^2^ test with the Benjamini–Hochberg method to detect DEGs (FDR < 0.05).

### 2.8. The KEGG Pathway Analysis of DEGs

Key metabolic pathways were manually checked using the KEGG Mapper [24]. A metabolic pathway diagram associated with the DEGs was drawn using PathVisio (ver. 3.3.0) [25].

## 3. Results

### 3.1. The Preparation of MBP-Fused KojR for Use in gSELEX

The predicted conformation of the MBP-KojR fusion protein confirmed that the location of the DNA binding domain (Zn(II)_2_Cys_6_ region, amino acids from the 15th to 58th of KojR) in the MBP fusion full-length KojR or MBP fusion KojR_1–70_ (70 amino acids on the N-terminal side of KojR), expressed from the DNA construct used in this study, is spatially distant from the MBP region (Supplementary Figure S1). The target proteins, expressed using a cell-free protein synthesis system with DNA constructs as templates, were preferentially produced at the designed size (Supplementary Figure S2).

### 3.2. The Genome-Wide Identification of KojR Binding Sites Using gSELEX-Seq

**Figure 1:**
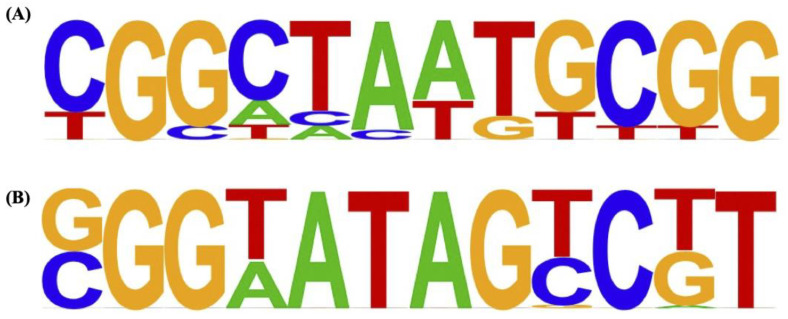
KojR binding motif detected using gSELEX-Seq. (**A**) Full-length KojR-binding motif (**B**) The binding motif of KojR_1–70_. *de novo* motif analysis of the KojR-binding site was performed using Homer (v4.11.1). The highest-ranked motif was indicated based on the criteria based on *p*-values in full-length KojR or KojR_1–70_.

The DNA pool, derived from the third selection round of gSELEX using either MBP-fused full-length KojR or MBP-fused KojR_1–70_, was sequenced using the Illumina NextSeq 550 system, and genome-wide KojR-binding sites were identified using bioinformatics analysis. The region detected as the peak was subjected to sequence motif analysis, and 5′-CGGCTAATGCGG-3′ was detected as a KojR-binding motif in the results of gSELEX-Seq using full-length MBP-fused KojR (Figure 1A). The sequence 5′-(G/C)GG(T/A)ATAG(T/C)C(T/G)T-3′ was identified as the binding motif in gSELEX-Seq using MBP-fused KojR_1–70_, although this sequence was somewhat less conserved compared to the sequence provided by the full-length KojR (Figure 1B).

Furthermore, when the regions detected as peaks were compared with the 1000 bp regions upstream of all genes in *A. oryzae*, 32 promoter regions were detected that might potentially bind to the MBP-fused full-length KojR (Table 1). Additionally, it was found that 16 genes possess promoter regions where the MBP-fused KojR_1–70_ region is likely to bind (Supplementary Table S2). The promoter region of *kojT* (AO090113000138) was detected in both cases using MBP-fused full-length KojR and MBP-fused KojR_1–70_.

### 3.3. The Identification of KojR Expression-Dependent DEGs Using RNA-Seq

**Figure 2:**
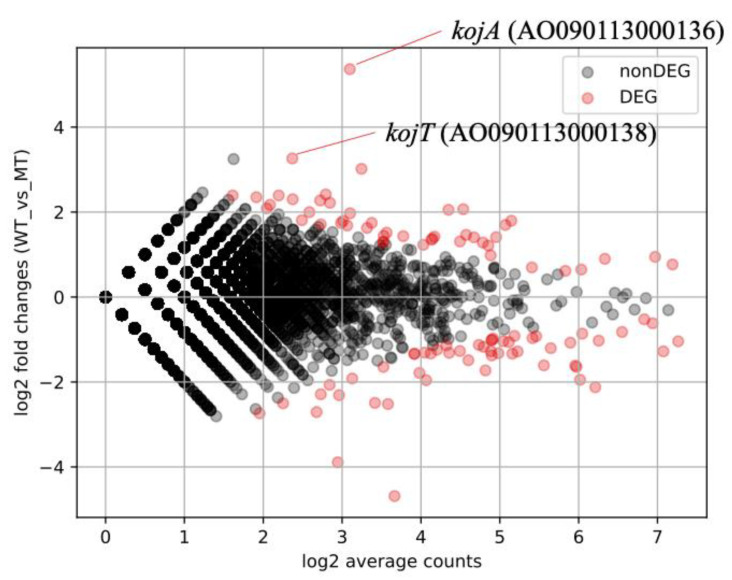
MA-plot based on RNA-Seq read data using *A. oryzae ΔkojR* and *A. oryzae* RIB40 strains. *X*-axis, average value of log_2_(average number of reads for each gene + 1) in *A. oryzae* RIB40 (WT) and *A. oryzae ΔkojR* (MT) strains; *Y*-axis, difference value of log_2_(average number of reads for each gene + 1) in WT and MT. FDR values were calculated through multiple testing with χ^2^ values, followed by correction applying the Benjamini-Hochberg method.

Initially, *A. oryzae ΔkojR* and RIB40 strains were cultured for RNA preparation for RNA-Seq. After culturing, the medium of *A. oryzae* RIB40 showed red coloration, indicating KA secretion, while no pigmentation was observed in the medium of *A. oryzae ΔkojR* (Supplementary Figure S3). To comprehensively identify genes dependent on KojR expression, total RNA extracted from *A. oryzae ΔkojR* and *A. oryzae* RIB40 was analyzed using nanopore sequencing. After processing the acquired sequence data of the 11,902 *A. oryzae* genes using various bioinformatics tools, we identified 100 genes whose expression levels varied significantly between *A. oryzae ΔkojR* and *A. oryzae* RIB40 strains (Figure 2 and Supplementary Table S3). AO090113000136 (*kojA*) showed the most significant differential expression among all analyzed genes, with a markedly higher expression in the RIB40 strain compared to the *ΔkojR* strain. AO090113000138 (*kojT*) also showed a significant increase in expression in the RIB40 strain compared with that of the *ΔkojR* strain. Notably, the number of genes with increased expression in the RIB40 strain was similar to the number of genes with decreased expression, compared to the *ΔkojR* strain (Figure 2). Certain genes, including AO090010000444, that encoded a predicted phosphofructokinase subunit protein, demonstrated notably higher expression in the *ΔkojR* strain compared to the RIB40 strain (Supplementary Table S3).

### 3.4. The Comprehensive Identification of the Target Genes of KojR Using Integrated Analysis-Based Binding Sites on A. oryzae Genome and DEGs Information

**Figure 3:**
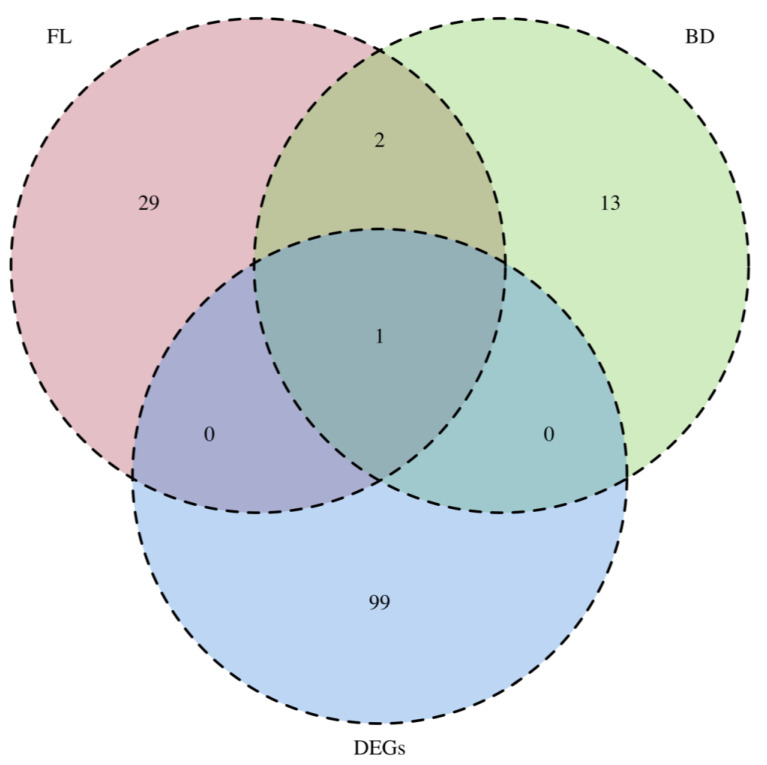
Venn diagram of the number of KojR-related genes obtained using gSELEX-Seq and RNA-Seq. FL, genes controlled by the candidate KojR-regulated promoters obtained using gSELEX with MBP-fused full-length KojR; BD, genes controlled by the candidate KojR-regulated promoters obtained using gSELEX with MBP-fused KojR_1–70_; DEGs, DEGs obtained using RNA-seq with *A. oryzae ΔkojR* and *A. oryzae* RIB40 strains.

The dataset of the genes with KojR binding sites in the promoter region obtained by performing gSELEX-Seq using the MBP-fused full length KojR or MBP-fused KojR_1–70_ and the dataset of KojR-dependent DEGs obtained by performing RNA-Seq with the *ΔkojR* strain were compared in an integrated manner (Figure 3). The intersections between the datasets obtained using gSELEX-Seq from MBP-fused KojR and MBP-fused KojR_1–70_ included AO090113000138 (*kojT*), AO090038000029, and AO090701000448. Furthermore, comparing the datasets obtained from gSELEX-Seq to the KojR-dependent DEG dataset showed that only AO090113000138 (*kojT*) was detected in the intersection (Figure 3).

## 4. Discussion

KojR, reported by Marui et al. in 2011, is a transcriptional regulator strongly related to KA synthesis in *A. oryzae* [6]. Although KojR has been predicted to bind to the CGG triplet, which is a typical target DNA sequence of this type because it possesses a Zn(II)_2_Cys_6_ zinc finger domain, the identification of the genome-wide binding site and target genes has not yet been reported. In the present study, we attempted to elucidate the regulatory mechanism underlying KA synthesis through a detailed functional analysis of KojR.

We comprehensively identified the KojR binding sites at the genomic level using gSELEX-Seq, which does not involve an intracellular TF–DNA binding reaction, such as chromatin immunoprecipitation, and rapidly and easily identifies the binding sites of the TF of interest. Previous approaches using gSELEX-Seq have used only the N-terminal region (including the DNA-binding domain) of the target TF that was prepared using an *E. coli* recombinant protein expression system as the MBP fusion protein [10,11]. Although this strategy, which uses only the N-terminal region of the target TF, allows for the efficient preparation of active MBP-fused TFs, the lack of a C-terminal region risks impairing the native intracellular functions such as stability and dimerization. In fact, gSELEX-Seq with AoXlnR_1—183_, the N-terminal region of AoXlnR, provided a DNA motif, which may be due to binding to the MBP-fused AoXlnR_1–183_ monomer [11]. 

To circumvent the problems associated with the restricted expression of full-length TFs in *E. coli* cells, this study employed a cell-free protein synthesis system to express the target TF. To evaluate this strategy, this system was used with MBP-fused full length KojR or MBP-fused KojR_1–70_ expression DNA constructs as templates. Subsequently, pronounced MBP-fused KojR expression was detected in the soluble fraction (Supplementary Figure S2). This result shows that gSELEX-Seq can be made more rapid and simpler by using a cell-free protein synthesis system as it circumvents the step where *E. coli* is cell cultured for expressing the recombinant protein, which is time-consuming and often limits the expression of active proteins from eukaryotes. Notably, MBP-fused full-length KojR was successfully expressed in the soluble fraction (Supplementary Figure S2). This result strongly reinforces the effectiveness of the strategy of using cell-free protein synthesis systems for exogenous protein expression, as this may overcome the problems associated with the lack of a C-terminal region described earlier. However, the expression level of MBP-fused full-length KojR was considerably lower than that of MBP-fused KojR_1–70_ (Supplementary Figure S2). The difference in the quantity of the synthesized products may be attributed to the preferential degradation of MBP-fused full-length KojR, as evidenced by the presence of several minor bands of smaller molecular weight in the soluble fraction after the expression of MBP-fused full-length KojR (Supplementary Figure S2).

Three rounds of gSELEX were performed with MBP-fused proteins prepared using a cell-free protein synthesis system, and the obtained DNA pool was applied to an Illumina NextSeq 550 to map the obtained read data to the *A. oryzae* genome. The analysis of the consensus sequence of the peak regions detected through this mapping clearly detected the CGG triplet in both MBP-fused full-length KojR and MBP-fused KojR_1–70_ (Figure 1). The results showed that the target sequences of the Zn(II)_2_Cys_6_-type TF KojR were preferentially detected, demonstrating the effectiveness of using gSELEX-Seq with KojR expressed using a cell-free protein synthesis system. Additionally, two CGG triplets were detected only for MBP-fused full-length KojR (Figure 1), suggesting that MBP-fused full-length KojR formed a dimer during the binding DNA selection process of gSELEX.

Next, we defined the promoter region as the segment 1000 bp upstream of the start codon of the *A. oryzae* gene and generated lists of genes that contained one or more peaks detected in response to KojR binding. The list contained 32 genes for the MBP-fused full-length KojR (Table 1) and 16 genes for the MBP-fused KojR_1–70_ (Supplementary Table S2). In both lists, *kojT*, a gene in the KA synthesis-related gene cluster, was identified as the top gene in terms of peak detection significance. These results strongly suggest the presence of a KojR-binding site in the promoter region of *kojT*. In fact, four CGGN_6_CGG sequences were densely distributed within the peak region of the *kojT* promoter on *A. oryzae* genome [13]. In contrast, no significant peak was detected in the *kojA* promoter region for KojR binding either with MBP-fused full-length KojR or MBP-fused KojR_1–70_ (Table 1 and Supplementary Table S2). The possibility that *in vitro* binding reaction conditions in gSELEX, such as KojR concentration, affect the binding of KojR to the *kojA* promoter region should not be completely excluded. However, at the very least, the results indicate that KojR binds preferentially to the *kojT* promoter region rather than to the *kojA* promoter region.

Previous studies have suggested the possibility that KojR binds to the promoter of *kojA* and induces the activation of *kojA* expression [6,26]; however, the gSELEX-Seq results in this study, in which the *kojA* promoter region was not selected as the KojR-binding region, make this hypothesis difficult to support. Chang et al. proposed the KojR-binding motif as a highly common sequence in the promoter region of *kojA* and *kojT*, CGRCTWAGYCG—which is relatively consistent with those of both full-length KojR and KojR_1–70_ shown in Figure 1. In fact, there is one site in the *A. oryzae kojA* promoter region that is exactly the same as the CGACTTTGCCG targeted by Chang et al. and two sites in the *A. oryzae kojT* promoter region that exactly match the proposed consensus sequence CGRCTWAGYCG. In addition, Chang et al. showed that KA synthesis is suppressed by introducing mutations in the sequences around the target binding motif in the *kojA* promoter region of *A. flavus* [8]. Although these results suggest that KojR binds to the *kojA* promoter and induces its expression in *A. flavus*, the possibility that the binding motif is the target site of the Zn(II)_2_Cys_6_ transcription factor involved in KA synthesis rather than KojR cannot be ruled out. Definitive conclusions regarding the presence or absence of KojR-binding sites in the *kojA* promoter may be obtained by preparing *kojA* promoters with various mutations at the KojR-binding site and quantitatively evaluating the binding affinity for KojR using surface plasmon resonance (SPR) or biolayer interferometry (BLI).

Notably, only three promoter regions (*kojT*, AO090038000029, and AO090701000448) were included in the intersection set when comparing the gene promoter sets detected using MBP-fused full-length KojR and MBP-fused KojR_1–70_ (Figure 3, Table 1, and Supplementary Table S2). AO090038000029 (*pcaC*) and AO090701000448 are predicted to retain peroxiredoxin and heme binding activity, respectively (Table 1, and Supplementary Table S2). It is difficult to directly correlate the function of these genes with KA synthesis at this time, since no marked changes in the expression of these genes were observed in the expression variation analysis using RNA from the fungi recovered after 3 days of culture (Supplementary Table S3). However, it is quite possible that the expression of these genes may change in a *kojR* expression-dependent manner under conditions after 3 days of culture, when KA synthesis is more advanced. On the other hand, the differences in the selected promoter regions may be attributed to the concentration of KojR in the *in vitro* binding reaction, and to the effects of KojR stability and dimerization, described earlier. Since conditions as close as possible to those of intact cells are more desirable, it would be appropriate to give priority to the promoter list obtained when using full-length KojR fused with MBP.

In parallel with the gSELEX-Seq analysis, we performed a comprehensive identification of KojR expression-dependent DEGs using RNA-Seq with the *ΔkojR* strain. *kojA* and *kojT* in the KA synthesis-related gene cluster were detected as DEGs, and their expression was reduced by the deletion of *kojR* (Figure 2 and Supplementary Table S3). This result is fully consistent with the previous report that the expression of *kojA* and *kojT* is suppressed in the *ΔkojR* strain, as shown with qRT-PCR analysis [6]. The results of this study obtained using RNA-Seq reinforce the association between KojR and the expression of *kojA* and *kojT*. 

Terabayashi et al. reported genes whose expression was induced in response to the increased synthesis of KA [5]. A comparison of the gene list with the DEGs obtained in this study revealed duplications of AO090120000112, AO090011000414, AO090001000237, and AO090003000018 in addition to *kojA* and *kojT*. Notably AO090001000237 is an ortholog of VeA, which is associated with secondary metabolism in *A. nidulans* (Supplementary Table S3). AO090011000414 was expected to exhibit glyceraldehyde-3-phosphate dehydrogenase activity during glycolysis (Supplementary Table S3). This finding reinforces the involvement of KojR in the metabolism of carbon sources in *A. oryzae*. Notably, Terabayashi et al. identified AO090011000414 as a gene whose expression is induced by increased KA synthesis, whereas we identified AO090011000414 as a DEG whose expression is higher than that in RIB40 in the *ΔkojR* strain in which KA synthesis is suppressed. This crucial difference suggests that the expression of genes involved in glycolysis is regulated intricately during secondary metabolite synthesis. Interestingly, the DEGs in this study included a GATA-type transcription factor, namely, AO090012000768 (*nsdD*) Supplementary Table S3), previously reported as potentially essential for KA synthesis [27].

In contrast, AO090012000623 (*nrtA*) [28], AO090003000489 (*laeA*) [29], AO090009000298 (*AoKap1*) [30], AO090120000102 (*AoKap2*) [31], AO090113000139 (*Aokap4*) [32], AO090009000515 (*Aokap5*) [33], AO090113000133 (*Aokap6*) [34], AO090005000940 (*AoZip2*) [35], AO090012000498 (*AozfA*) [36], AO090003001186 (*KpeA*) [27], AO090020000046 (*lreA*) [27], and AO090009000320 (*creB*) [27], which may be related to KA synthesis according to previous reports, were not included in the DEGs in this study. Particularly, Chen et al. reported a decreased expression of *Aokap6* owing to *kojR* deletion; however, the degree of change was relatively low [34]. Factors that contribute to the lack of observed expressional variations associated with the deletion of KojR in the genes described above include differences in sensitivity accompanied by analysis conditions, timing of RNA extraction and incubation time, and the possibility that KojR plays a role further downstream in the KA synthesis pathway.

The result of the Venn diagram of the number of KojR-related genes obtained from gSELEX-Seq and RNA-Seq strongly suggests that *kojT*, which has a KojR binding site in its promoter region and shows a KojR-dependent increase in expression, may be under the direct control of KojR (Figure 3). It should be noted that only *kojT* was detected at the intersection obtained from the gSELEX-Seq and KojR-dependent DEG dataset. DEGs for which no KojR binding sites were detected in the promoter region may be indirectly regulated by KojR. On the other hand, none of the genes identified by gSELEX-Seq showed significant KojR-dependent changes in expression levels, except for *kojT*, which may be mainly due to the culture time of *A. oryzae* for RNA extraction described above. It has been shown that KA, a secondary metabolite, is not secreted into the medium in *A. oryzae* in the early stage of culture, but its secretion increases linearly after a certain period of culture [5,6]. Although KA synthesis in the RIB40 culture solution was clearly observed in this study (Supplement Figure S3), the culture period was 3 days. This culture period may have been insufficient to clearly observe changes in the expression levels of genes associated with KA synthesis. Based on this hypothesis, there may be additional potential and direct regulators of KojR as well as *kojT* in the genes detected using gSELEX-Seq. Comparing the expression levels of all genes in *A. oryzae* over time would provide a more comprehensive identification of genes that are directly regulated by KojR.

**Figure 4:**
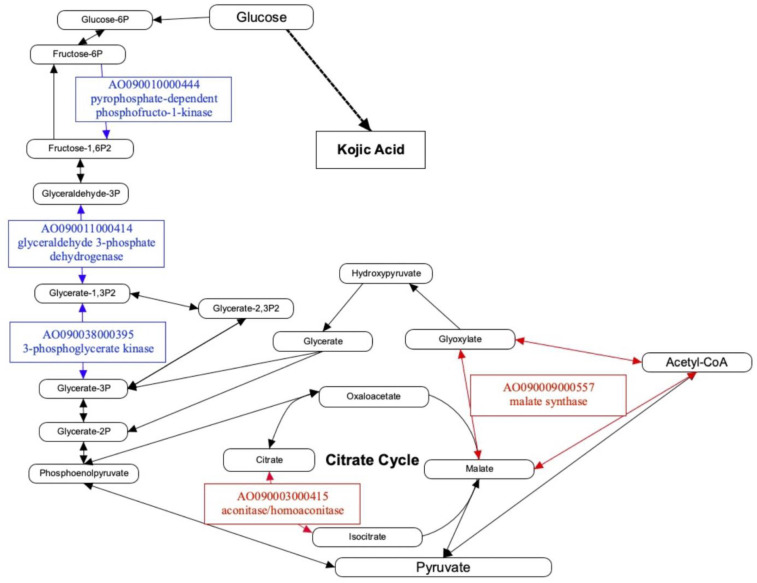
Mapping of differentially expressed genes (DEGs) associated with *kojR* expression to *A. oryzae* carbon metabolic pathway. Red, DEG whose expression levels in *A. oryzae* RIB40 are higher than that in *ΔkojR* strain; blue, DEG with lower expression levels in *A. oryzae* RIB40 than that in *ΔkojR* strain. A metabolic map was created using the PathVisio software (Ver. 3.3.0) [25] based on the carbon metabolism (aor01200) and glycolysis/gluconeogenesis (aor00010) of *A. oryzae* in KEGG [24].

Finally, metabolic pathway mapping using the KEGG pathway [24] was performed to evaluate the role of the 100 DEGs, which were detected using RNA-Seq analysis, in the carbon-source metabolic pathway in *A. oryzae* cells. In total, five DEGs (AO090010000444, AO090011000414, AO090038000395, AO090003000415, and AO090009000557) were mapped onto the carbon metabolism and glycolysis pathways in *A. oryzae* (Figure 4). AO090010000444, AO090011000414, and AO090038000395 are involved in the glycolytic system and were upregulated in the *ΔkojR* strain. In contrast, AO090003000415 and AO090009000557 were involved in the citrate cycle and were downregulated in the *ΔkojR* strain. The mapping results suggest that KojR may be involved in the inhibition of glycolysis but contribute to TCA cycle activation (Figure 4). However, since no KojR binding sites were detected in the promoter regions of the genes analyzed using gSELEX-Seq (Table 1 and Supplementary Table S2), it is possible that these DEGs are indirectly regulated by KojR. Taken together, these results suggested that KojR is indirectly but closely related to glucose-initiated KA synthesis.

## 5. Conclusions

In this study, we performed a comprehensive search for KojR target genes and showed that *kojT* is directly regulated by KojR. Additionally, we identified a group of genes associated with KojR expression and showed that KojR expression is closely related to the expression of a group of enzymes involved in C-source metabolism. We believe that the findings of this study will contribute greatly to the elucidation of the KA biosynthesis mechanism in *A. oryzae* using C-sources such as glucose.

## Figures and Tables

**Table 1:** Genes containing full-length KojR binding region in 1000 bp upstream of the initiation codon.

Peak Region	Gene ID	Original Description
Chr5_A_oryzae_RIB40:4372480-4372692	AO090113000138	Putative transporter; present in the kojic acid biosynthetic gene cluster
Chr6_A_oryzae_RIB40:3584694-3584906	AO090038000029	Has domain(s) with predicted peroxiredoxin activity and role in oxidation-reduction process
Chr5_A_oryzae_RIB40:1165262-1165474	AO090701000448	Has domain(s) with predicted heme binding activity
Chr5_A_oryzae_RIB40:643370-643582	AO090701000654	Protein of unknown function
Chr5_A_oryzae_RIB40:1158260-1158472	AO090701000450	Has domain(s) with predicted catalytic activity, hydrolase activity, and role in metabolic process
Chr1_A_oryzae_RIB40:5569621-5569833	AO090005000336	Has domain(s) with predicted sequence-specific DNA binding RNA polymerase II transcription factor activity, zinc ion binding activity, and role in regulation of transcription, DNA-templated and nucleus localization
Chr7_A_oryzae_RIB40:229700-229912	AO090011000078	Ortholog of *A. niger* CBS 513.88: An01g06430, *A. versicolor*: Aspve1_0082366, *A. niger* ATCC 1015: 36036-mRNA, and *A. zonatus*: Aspzo1_0026572
Chr1_A_oryzae_RIB40:4941497-4941709	AO090005000571	Has domain(s) with predicted NAD binding, oxidoreductase activity, activity on the CH-OH group of donors, NAD or NADP as acceptor activity, and role in oxidation-reduction process
Chr2_A_oryzae_RIB40:4505321-4505533	AO090003000890	Protein of unknown function
Chr4_A_oryzae_RIB40:4149411-4149623	AO090102000151	Ortholog(s) have ubiquitin–protein transferase activity, have a role in protein import into peroxisome matrix and are an integral component of peroxisomal membrane localization
Chr1_A_oryzae_RIB40:6476509-6476721	AO090308000007	Protein of unknown function
Chr3_A_oryzae_RIB40:391752-391964	AO090023000155	Protein of unknown function
Chr6_A_oryzae_RIB40:3081726-3081938	AO090038000221	Protein of unknown function
Chr7_A_oryzae_RIB40:1923126-1923338	AO090011000751	Protein of unknown function
Chr2_A_oryzae_RIB40:5473553-5473765	AO090003001242	Has domain(s) with predicted ATP binding and inositol pentakisphosphate 2-kinase activity
Chr6_A_oryzae_RIB40:1409858-1410070	AO090020000161	Ortholog of *A. nidulans* FGSC A4: AN6458, *A. fumigatus* Af293: Afu3g07420, *A. niger* CBS 513.88: An02g10960, An01g01420, An12g05390, and *A. oryzae* RIB40: AO090005000921, AO090003001427
Chr6_A_oryzae_RIB40:1409858-1410070	AO090020000162	Has domain(s) with predicted UDP-N-acetylmuramate dehydrogenase activity, flavin adenine dinucleotide binding, oxidoreductase activity, and activity on the CH-OH group of donors
Chr6_A_oryzae_RIB40:4172129-4172341	AO090138000010	Has domain(s) with predicted 2-dehydropantoate 2-reductase activity, NADP binding, coenzyme binding, oxidoreductase activity, activity on the CH-OH group of donors, and NAD or NADP as acceptor activity
Chr5_A_oryzae_RIB40:1114034-1114246	AO090701000470	Ortholog of *A. fumigatus* Af293: Afu2g16985, *A. wentii*: Aspwe1_0171101, *A. clavatus* NRRL 1: ACLA_075940, and *A. zonatus*: Aspzo1_0015705
Chr3_A_oryzae_RIB40:1794701-1794913	AO090023000683	Protein of unknown function
Chr6_A_oryzae_RIB40:3558859-3559071	AO090038000040	Has domain(s) with predicted catalytic activity, catechol 1,2-dioxygenase activity, ferric iron binding, iron ion binding and oxidoreductase activity, in addition to other activities
Chr1_A_oryzae_RIB40:3825460-3825672	AO090005000971	Has domain(s) with a predicted role in the biosynthetic process
Chr5_A_oryzae_RIB40:4076380-4076592	AO090113000012	Ortholog of *A. nidulans* FGSC A4: AN6909/BEST2, *A. niger* CBS 513.88: An14g05100, *A. wentii*: Aspwe1_0153378, *A. sydowii*: Aspsy1_0054771, and *A. terreus* NIH2624: ATET_06151
Chr7_A_oryzae_RIB40:2355701-2355913	AO090011000905	Ortholog(s) have sequence-specific DNA binding transcription factor activity
Chr7_A_oryzae_RIB40:2498610-2498822	AO090011000954	Ortholog(s) have cytosol localization
Chr8_A_oryzae_RIB40:1654282-1654494	AO090010000667	Has domain(s) with predicted iron ion binding and oxidoreductase activity; role in fatty acid biosynthetic process and oxidation-reduction process
Chr3_A_oryzae_RIB40:244139-244351	AO090023000096	Has domain(s) with predicted catalytic activity, coenzyme binding activity, and role in cellular metabolic process
Chr8_A_oryzae_RIB40:1372821-1373033	AO090010000775	Ortholog(s) have UDP-N-acetylglucosamine transmembrane transporter activity and role in UDP-N-acetylglucosamine transport, UDP-glucose transport, fungal-type cell wall chitin biosynthetic process, and transmembrane transport
Chr1_A_oryzae_RIB40:3919266-3919478	AO090005000937	Ortholog of *A. nidulans* FGSC A4: AN1323 and *A. flavus* NRRL 3357: AFL2T_00913
Chr6_A_oryzae_RIB40:442735-442947	AO090020000539	Ortholog(s) have Golgi apparatus, endoplasmic reticulum localization
Chr8_A_oryzae_RIB40:2804269-2804481	AO090010000224	Ortholog(s) have role in ethanol metabolic process and mitochondrial inner membrane localization
Chr8_A_oryzae_RIB40:2804269-2804481	AO090010000223	40S ribosomal protein S2-like protein; predominantly expressed in the hyphal tip region

Original description is based on information provided by the Comprehensive *Aspergillus oryzae* Genome Database (CAoGD) (https://nribf21.nrib.go.jp/CAoGD/ (accessed on 12 April 2023)).

## Data Availability

The reported nucleotide sequence data are available in the DDBJ Sequenced Read Archive under the accession numbers PRJDB16474 and PRJDB16475.

## Supplementary Materials

The following supporting information can be downloaded at: https://www.mdpi.com/article/10.3390/jof10020113/s1, Supplementary Figure S1: Predicted 3D structure of MBP-fused KojR used in gSELEX [37]; Supplementary Figure S2: SDS-PAGE analysis of MBP fusion proteins containing full-length KojR or KojR DNA binding domain synthesized by cell-free protein synthesis system; Supplementary Figure S3: Evaluation of KA synthesis in cultures of *A. oryzae* RIB40 and *ΔkojR* strains for RNA preparation; Supplementary Table S1: Oligonucleotides used in this study; Supplementary Table S2: Genes containing KojR_1–70_ binding region in 1000 bp upstream of the initiation codon; Supplementary Table S3: DEGs detected using RNA-Seq in *A. oryzae ΔkojR* and RIB40 strains.
