# Supplementary material for: The Identification of a Target Gene of the Transcription Factor KojR and Elucidation of Its Role in Carbon Metabolism for Kojic Acid Biosynthesis in Aspergillus oryzae
Source: J Fungi (Basel). 2024 Jan 30;10(2):113. doi: 10.3390/jof10020113 (PMC10890517; doi:10.3390/jof10020113)
Supplement: Supplementary file 1 [file jof-10-00113-s001.zip › Supplementary Table S1. Oligonucleotides used in this study.pdf]

Supplementary Table S1. Oligonucleotides used in this study.

Name	Sequence
HO-kojR-F	5′-AGGATTTCAG AATTCATGTC GTTGAATACC GACGATTC-3′
HO-kojR-R	5′-TAGAGGATCT TATTATTATC TATATCTCTG ACCACCTG-3′
HO-kojR-BD-R	5′-TAGAGGATCT TATTATTATT CTTCCTCGCT GGGCCGTG-3′
PMAL-IFC-F1	5′-TAATAAGATC CTCTAGAGTC GACCTGC-3′
PMAL-IFC-R1	5′-GAATTCTGAA ATCCTTCCCT CGATCCC-3′
kojR-F	5′-ATGTCGTTGA ATACCGACGA TTCC-3′
kojRFull-R	5′-TTATCTATAT CTCTGACCAC CTGC-3′
kojRBD-R	5′-TTCTTCCTCG CTGGGCCG-3′
gibson_Full-F	5′-GGTGGTCAGA GATATAGATA ATGATCCGGC TGCTAACAAA G-3′
gibson_BD-F	5′-GCCCAGCGAG GAAGAATGAT CCGGCTGCTA ACAAAG-3′
gibson_kojR-R	5′-GTCGGTATTC AACGACATGA ATTCTGAAAT CCTTCCCTCG-3′
F1-primer	5′-ATCTCGATCC CGCGAAATTA ATACG-3′
R1-primer	5′-TCCGGATATA GTTCCTCCTT TCAG-3′
Nextera-Read1	5′-TCGTCGGCAG CGTCAGATGT GTATAAGAGA CAG-3′
Nextera-Read2	5′-GTCTCGTGGG CTCGGAGATG TGTATAAGAG ACAG-3′
ppAsAC9gwA_F7	5′-GATTCCGGTC GGATAAGGAC CGTTTTAGAG CTAGAAATAG CAAGTTAAAA TA-3′
ppAsAC9gwA_R7	5′-GGTCCTTATC CGACCGGAAT CACTTGTTCT TCTTTACAAT GATTTATTTA CC-3′
kojR_CP_F	5′-TTCCGACTGC CCGATAACCT TGTAC-3′
kojR_CP_R	5′-TGGCCTCCAT GTGTTCTTGG TTAGC-3′
kojR_seq	5′-CCCGAACCCC CCACTG-3′
