# Supplementary material for: The Identification of a Target Gene of the Transcription Factor KojR and Elucidation of Its Role in Carbon Metabolism for Kojic Acid Biosynthesis in Aspergillus oryzae
Source: J Fungi (Basel). 2024 Jan 30;10(2):113. doi: 10.3390/jof10020113 (PMC10890517; doi:10.3390/jof10020113)
Supplement: Supplementary file 1 [file jof-10-00113-s001.zip › Supplementary Table S2. Genes containing KojR1í¬70 binding region in 1000 bp upstream of the initiation codon.pdf]

Supplementary Table S2. Genes containing KojR$_{1—70}$ binding region in 1000 bp upstream of the initiation codon.

Peak Region	Gene ID	Original description
Chr5_A_oryzae_RIB40:4372485-4372683	AO090113000138	Putative transporter; present in the kojic acid biosynthetic gene cluster
Chr5_A_oryzae_RIB40:1165268-1165466	AO090701000448	Has domain(s) with predicted heme binding activity
Chr3_A_oryzae_RIB40:2038063-2038261	AO090023000783	Has domain(s) with predicted catalytic activity, nitronate monooxygenase activity and role in oxidation-reduction process
Chr3_A_oryzae_RIB40:3419755-3419953	AO090026000613	Ortholog(s) have ATP binding, ATPase activity, metallopeptidase activity, role in protein complex assembly, proteolysis, signal peptide processing and m-AAA complex, mitochondrial inner boundary membrane localization
Chr2_A_oryzae_RIB40:2312210-2312408	AO090003000082	Ortholog of *Aspergillus flavus* NRRL 3357: AFL2T_02865
Chr5_A_oryzae_RIB40:3813614-3813812	AO090120000422	Ortholog(s) have DNA-directed DNA polymerase activity, role in DNA replication initiation, telomere capping and alpha DNA polymerase:primase complex, cytosol, nuclear envelope localization
Chr5_A_oryzae_RIB40:3813614-3813812	AO090120000421	Ortholog(s) have phosphatidylinositol transporter activity, role in cellular response to drug, phospholipid biosynthetic process, phospholipid transport, sterol biosynthetic process and lipid particle, plasma membrane localization
Chr6_A_oryzae_RIB40:953-1151	AO090020000719	Ortholog of *A. nidulans* FGSC A4: AN2370, AN10160, AN0323, *A. fumigatus* Af293: Afu1g02440, Afu4g02760, *A. niger* CBS 513.88:

		An01g05750, An12g09350, An13g03290 and *A. oryzae* RIB40: AO090005000852
Chr8_A_oryzae_RIB40:266490-266688	AO090103000394	Has domain(s) with predicted transmembrane transporter activity, role in transmembrane transport and integral component of membrane localization
Chr6_A_oryzae_RIB40:3584722-3584920	AO090038000029	Has domain(s) with predicted peroxiredoxin activity and role in oxidation-reduction process
Chr1_A_oryzae_RIB40:485924-486122	AO090009000181	Protein of unknown function
Chr3_A_oryzae_RIB40:4007711-4007909	AO090026000392	Ortholog(s) have mitochondrion localization
Chr4_A_oryzae_RIB40:4473364-4473562	AO090102000028	Has domain(s) with predicted ATP binding, ATPase activity, ATPase activity, coupled to transmembrane movement of substances, nucleoside-triphosphatase activity, nucleotide binding activity and role in transport
Chr3_A_oryzae_RIB40:1724542-1724740	AO090023000653	Ortholog(s) have ferrous iron binding activity, role in peptidyl-diphthamide biosynthetic process from peptidyl-histidine and cytosol, nucleus localization
Chr2_A_oryzae_RIB40:624894-625092	AO090001000256	Has domain(s) with predicted DNA binding, zinc ion binding activity, role in transcription, DNA-templated and nucleus localization
Chr3_A_oryzae_RIB40:1546518-1546716	AO090023000583	Ortholog of *A. nidulans* FGSC A4: AN7052, *A. fumigatus* Af293: Afu4g09510, Afu4g03945, *A. niger* CBS 513.88: An14g00940, An07g04990, An04g08460 and *A. oryzae* RIB40: AO090011000324, AO090005000036

Original description is based on information provided by the Comprehensive *Aspergillus oryzae*

Genome Database (CAoGD) (https://nribf21.nrib.go.jp/CAoGD/ (accessed on 12 April 2023)).
