# Supplementary material for: The Identification of a Target Gene of the Transcription Factor KojR and Elucidation of Its Role in Carbon Metabolism for Kojic Acid Biosynthesis in Aspergillus oryzae
Source: J Fungi (Basel). 2024 Jan 30;10(2):113. doi: 10.3390/jof10020113 (PMC10890517; doi:10.3390/jof10020113)
Supplement: Supplementary file 1 [file jof-10-00113-s001.zip › Supplementary Table S3. DEGs detected using RNA-Seq in A. oryzae añkojR and RIB40 strains.pdf]

Supplementary Table S3. DEGs detected using RNA-Seq in *A. oryzae ΔkojR* and RIB40 strains.

Gene ID	Up or Down	FDR	Original description
AO090113000136	+	9.06E-33	FAD-dependent oxidoreductase; present in the kojic acid biosynthetic gene cluster
AO090005000583	-	1.84E-31	Ortholog of *A. nidulans* FGSC A4: AN1549, *A. fumigatus* Af293: Afu8g05600, *A. niger* CBS 513.88: An16g07080, *Aspergillus wentii*: Aspwe1_0187131 and *Aspergillus sydowii*: Aspsy1_0086712
AO090023000589	-	3.79E-23	Ortholog of *A. niger* CBS 513.88: An11g03810, *Neosartorya fischeri* NRRL 181: NFIA_063740, NFIA_095150 and *Aspergillus wentii*: Aspwe1_0038630, Aspwe1_0045728
AO090026000254	-	3.79E-23	Ortholog(s) have pyruvate dehydrogenase (acetyl-transferring) kinase activity, role in carbon utilization, peptidyl-serine phosphorylation and mitochondrion localization
AO090009000166	-	9.46E-22	Ortholog of *A. nidulans* FGSC A4: AN3724, *A. fumigatus* Af293: Afu6g12460, *A. niger* CBS 513.88: An06g01620, *Aspergillus wentii*: Aspwe1_0033694 and *Aspergillus sydowii*: Aspsy1_0044118
AO090012000670	-	3.21E-16	Ortholog of *A. fumigatus* Af293: Afu3g14260, *A. niger* CBS 513.88: An09g04240, *Neosartorya fischeri* NRRL 181: NFIA_063040 and *Aspergillus wentii:* Aspwe1_0038047
AO090010000443	-	4.75E-16	Ortholog of *Aspergillus flavus* NRRL 3357: AFL2T_11611
AO090010000444	-	2.64E-15	Predicted phosphofructokinase subunit with role in glycolysis; transcription is repressed

			by glucose; upregulated under hypoxic growth conditions
AO090102000620	+	7.95E-15	Ortholog(s) have ATPase activity, coupled, chaperone binding, unfolded protein binding activity
AO090003000546	+	7.95E-15	Ortholog(s) have DNA binding, bending, RNA polymerase I transcription factor binding and RNA polymerase I transcription factor recruiting transcription factor activity, more
AO090023000918	+	9.34E-13	Ortholog of *A. nidulans* FGSC A4: AN4379, *A. fumigatus* Af293: Afu4g06670/aspf7, *Neosartorya fischeri* NRRL 181: NFIA_109540 and *Aspergillus wentii*: Aspwe1_0023301
AO090120000112	+	1.92E-12	Ortholog(s) have IgE binding, thioredoxin peroxidase activity and extracellular region, peroxisome localization
AO090701000343	-	2.40E-11	Ortholog of *A. nidulans* FGSC A4: AN10977, A. fumigatus Af293: Afu2g00790, *Aspergillus wentii*: Aspwe1_0052113 and *Aspergillus sydowii*: Aspsy1_0048752
AO090003001182	+	2.89E-11	Ortholog of *A. nidulans* FGSC A4: AN2000/ubi4, A. fumigatus Af293: Afu4g10350/ubiD, A. niger CBS 513.88: An04g06510, *Aspergillus wentii*: Aspwe1_0047475 and *Aspergillus sydowii*: Aspsy1_0053937
AO090103000022	+	3.62E-11	Ortholog(s) have choline:oxygen 1-oxidoreductase activity, role in choline catabolic process and intracellular localization
AO090701000742	+	4.94E-11	Ortholog(s) have role in cellular response to biotic stimulus, cellular response to farnesol, cellular response to heat, cellular response to

			hydrogen peroxide and cellular response to starvation, more
AO090038000465	-	1.67E-09	Has domain(s) with predicted protein disulfide oxidoreductase activity
AO090012000495	+	3.53E-08	Ortholog(s) have role in DNA methylation, global genome nucleotide-excision repair, mitotic spindle assembly checkpoint, rRNA transcription, regulation of DNA methylation, sexual sporulation resulting in formation of a cellular spore
AO090020000540	+	1.36E-07	Ortholog(s) have cytoplasm localization
AO090003000458	-	1.71E-07	Ortholog(s) have uracil DNA N-glycosylase activity and nucleus localization
AO090011000201	-	2.17E-07	Ortholog of *A. nidulans* FGSC A4: AN3215, AN7166, AN8609, *A. fumigatus* Af293: Afu3g14210, Afu4g03360, Afu4g12510 and *A. niger* CBS 513.88: An09g04200, An14g01840
AO090113000138	+	4.99E-07	Putative transporter; present in the kojic acid biosynthetic gene cluster
AO090003000457	-	1.59E-06	Ortholog(s) have role in RNA metabolic process and cytosol, nucleus localization
AO090026000547	-	2.31E-06	Ortholog(s) have 3'-tyrosyl-DNA phosphodiesterase activity, role in single strand break repair and nucleus localization
AO090120000183	-	3.56E-06	Protein of unknown function
AO090009000715	+	3.56E-06	Ortholog(s) have role in maturation of SSU-rRNA from tricistronic rRNA transcript (SSU-rRNA, 5.8S rRNA, LSU-rRNA) and 90S preribosome, cytosol, extracellular region localization
AO090003000018	+	4.12E-06	Hsp30-like protein; positively regulated by XlnR
AO090003000919	-	1.22E-05	Ortholog of *A. fumigatus* Af293: Afu1g04300, *A. niger* CBS 513.88:

			An01g03130, *A. oryzae* RIB40: AO090701000446, *Aspergillus wentii*: Aspwe1_0025379 and *Aspergillus terreus* NIH2624: ATET_06531
AO090038000286	-	1.33E-05	Ortholog of *A. nidulans* FGSC A4: AN9073, AN1160, A. fumigatus Af293: Afu1g11220, Afu7g02460, *A. niger* CBS 513.88: An12g00140 and *Aspergillus wentii*: Aspwe1_0173751
AO090102000506	-	1.63E-05	Protein of unknown function
AO090012000521	-	1.69E-05	Ortholog(s) have intracellular localization
AO090038000287	+	1.86E-05	Ortholog(s) have guanyl-nucleotide exchange factor activity, translation elongation factor activity, role in translational elongation and cell surface, cytosol, eukaryotic translation elongation factor 1 complex, ribosome localization
AO090038000395	-	1.86E-05	Predicted 3-phosphoglycerate kinase with role in glycolysis
AO090003000947	+	4.76E-05	Ortholog(s) have role in protein ubiquitination, ribosome biogenesis and cytosol, extracellular region, membrane, mitochondrion, nucleolus localization
AO090005001291	+	5.59E-05	Ortholog(s) have role in rRNA processing and 90S preribosome, box C/D snoRNP complex, small-subunit processome localization
AO090012000821	-	6.34E-05	Ortholog of *A. nidulans* FGSC A4: AN10356, AN0435, *A. fumigatus* Af293: Afu1g04510, Afu3g13080, *A. niger* CBS 513.88: An01g03600 and *A. oryzae* RIB40: AO090003000870
AO090102000351	-	7.92E-05	Putative membrane protein
AO090102000336	-	0.000123	Has domain(s) with predicted catalytic activity

AO090012000496	+	0.000151	Histone
AO090009000288	+	0.000288	Ortholog(s) have histone demethylase activity (H3-K9 specific), nucleosome binding activity and role in antisense RNA transcription, histone H3-K9 demethylation, positive regulation of transcription, DNA-templated, regulation of meiosis
AO090009000557	+	0.000288	Malate synthase; upregulated under hypoxic growth conditions
AO090120000322	-	0.0003	DNA ligase IV homolog involved in the final step of nonhomologous end joining
AO090003000984	-	0.000305	Has domain(s) with predicted heme binding, iron ion binding, oxygen binding activity and role in oxygen transport
AO090011000518	+	0.000384	Ortholog of *A. nidulans* FGSC A4: AN4622, *A. fumigatus* Af293: Afu2g02380, *A. niger* CBS 513.88: An07g06420, *Aspergillus wentii*: Aspwe1_0168908 and *Aspergillus sydowii*: Aspsy1_0155773
AO090010000221	-	0.000384	Bacterial rhodopsin family G-protein coupled receptor-like protein
AO090003000150	+	0.000558	Ortholog of *A. nidulans* FGSC A4: AN3310, AN1449, AN7654, *A. fumigatus* Af293: Afu2g01140, Afu3g00880, Afu8g04370 and *A. niger* CBS 513.88: An15g07790, An10g00430
AO090701000554	-	0.000558	Heat shock protein
AO090005001207	+	0.000708	Ortholog of *A. nidulans* FGSC A4: AN0860, *A. fumigatus* Af293: Afu1g15260, *A. niger* CBS 513.88: An01g13370, *A. oryzae* RIB40: AO090103000449 and *Aspergillus wentii*: Aspwe1_0111296
AO090026000179	-	0.000963	Ortholog of *A. nidulans* FGSC A4: AN6380, *A. fumigatus* Af293: Afu2g13930, *A. niger* CBS 513.88: An02g05120, *Neosartorya*

			fischeri NRRL 181: NFIA_089090 and *Aspergillus versicolor*: Aspve1_0052622
AO090009000459	+	0.00108	Ortholog(s) have RNA polymerase II transcription factor binding transcription factor activity involved in negative regulation of transcription, more
AO090020000043	+	0.00112	Has domain(s) with predicted sequence-specific DNA binding RNA polymerase II transcription factor activity, zinc ion binding activity, role in regulation of transcription, DNA-templated and nucleus localization
AO090020000512	-	0.00246	Protein of unknown function
AO090003001244	-	0.0026	Has domain(s) with predicted phosphoric diester hydrolase activity and role in lipid metabolic process
AO090023000141	+	0.00276	Has domain(s) with predicted hydrolase activity
AO090011000309	-	0.00313	Ortholog of *A. nidulans* FGSC A4: AN7139, *A. fumigatus* Af293: Afu4g03730, *A. niger* CBS 513.88: An14g01430, *Aspergillus wentii*: Aspwe1_0030342 and *Aspergillus sydowii*: Aspsy1_0034590
AO090005000428	+	0.0033	Ortholog(s) have ATPase activity, translation elongation factor activity, role in translational elongation and cell surface, cytosolic ribosome, plasma membrane, yeast-form cell wall localization
AO090701000206	+	0.00353	4-aminobutyrate transaminase, GABA transaminase; predominantly expressed in the basal region of hyphae
AO090012000768	+	0.00353	GATA-type transcription factor
AO090009000117	-	0.00353	Ortholog(s) have hydrolase activity, acting on glycosyl bonds, transferase activity, transferring glycosyl groups activity and role in carbohydrate metabolic process

AO090009000405	+	0.00353	Ortholog(s) have ATP:ADP antiporter activity
AO090002000060	-	0.00396	Ortholog(s) have cytochrome-c oxidase activity, role in aerobic respiration, mitochondrial electron transport, cytochrome c to oxygen and mitochondrial respiratory chain complex IV localization
AO090026000812	+	0.00439	Ortholog of *A. fumigatus* Af293: Afu7g04020, *A. niger* CBS 513.88: An13g00480, *Aspergillus wentii*: Aspwe1_0429172, *Aspergillus sydowii*: Aspsy1_0154048 and *Aspergillus terreus* NIH2624: ATET_04283
AO090103000456	-	0.00527	Has domain(s) with predicted N,N-dimethylaniline monooxygenase activity, NADP binding, flavin adenine dinucleotide binding activity and role in oxidation-reduction process
AO090023000570	+	0.00527	Glutathione S-transferase; upregulated in *A. oryzae* and *A. nidulans* under hypoxic growth conditions
AO090003000805	+	0.00557	Ortholog(s) have large ribosomal subunit rRNA binding activity, role in ribosomal large subunit assembly and 90S preribosome, cytosol, preribosome, large subunit precursor localization
AO090011000266	-	0.00572	Protein of unknown function
AO090038000281	-	0.00642	Ortholog of *A. nidulans* FGSC A4: AN8776, *A. niger* CBS 513.88: An08g03560, *Aspergillus wentii*: Aspwe1_0508022, *Aspergillus sydowii*: Aspsy1_0035009 and *Aspergillus terreus* NIH2624: ATET_00334
AO090003000922	+	0.00865	Ca^{2+}/H^{+} antiporter family protein
AO090005000278	-	0.00905	Protein of unknown function

AO090005001030	-	0.00955	Ortholog of *Aspergillus flavus* NRRL 3357: AFL2T_00992
AO090023000571	+	0.00967	Has domain(s) with predicted flavin adenine dinucleotide binding, oxidoreductase activity, acting on CH-OH group of donors activity and role in oxidation-reduction process
AO090003000043	+	0.00969	Ortholog of *A. nidulans* FGSC A4: AN5764, *A. fumigatus* Af293: Afu6g06670, *A. niger* CBS 513.88: An18g06390, *Aspergillus wentii*: Aspwe1_0170253 and *Aspergillus sydowii*: Aspsy1_0030199
AO090003000631	+	0.0109	Ortholog(s) have glutathione peroxidase activity, glutathione transferase activity and role in cellular response to metal ion, cellular response to xenobiotic stimulus
AO090001000237	+	0.0113	Ortholog of *A. nidulans* VeA, a global gene regulator involved in light-sensitive control of differentiation and secondary metabolism; positively regulates penicillin production of *A. oryzae*
AO090005001622	-	0.0152	Ortholog of *A. nidulans* FGSC A4: AN1378, *A. fumigatus* Af293: Afu1g09030, *A. niger* CBS 513.88: An08g00540, *Aspergillus wentii*: Aspwe1_0024518 and *Aspergillus sydowii*: Aspsy1_0138364
AO090023000516	+	0.0163	Ortholog(s) have cytosol, nucleus localization
AO090005001117	-	0.0177	Ortholog(s) have fatty acid alpha-hydroxylase activity, role in cellular response to cadmium ion, detoxification of cadmium ion, mannosyl-inositol phosphorylceramide metabolic process and endoplasmic reticulum, membrane localization

AO090026000184	-	0.0177	Has domain(s) with predicted calcium ion binding, calcium-dependent phospholipid binding activity
AO090020000513	-	0.0181	Has domain(s) with predicted N-acetyltransferase activity
AO090011000215	-	0.0181	bHLH transcription factor with a role in hyphal morphology, asexual conidiospore formation, and the promotion of sclerotial production
AO090102000352	-	0.0181	Ortholog(s) have cytosol, mitotic spindle pole body, nucleus localization
AO090011000414	-	0.0186	Ortholog(s) have glyceraldehyde-3-phosphate dehydrogenase (NAD^+) (phosphorylating) activity and extracellular region, intracellular, membrane localization
AO090002000050	+	0.0202	Ortholog of *A. nidulans* FGSC A4: AN20013 and *A. fumigatus* Af293: AfuMt00070
AO090003001054	-	0.0204	Ortholog(s) have Rpd3L complex, Rpd3L-Expanded complex, Rpd3S complex, cytosol localization
AO090011000118	+	0.0228	Ribonuclease T1; secreted protein
AO090003000575	-	0.026	Ortholog(s) have role in cellular response to drug, secondary metabolite biosynthetic process
AO090701000169	-	0.0261	Ortholog of *A. nidulans* FGSC A4: AN2183, *A. fumigatus* Af293: Afu6g03800, *A. niger* CBS 513.88: An15g01720, *Aspergillus wentii*: Aspwe1_0035996 and *Aspergillus sydowii*: Aspsy1_0084971
AO090020000517	+	0.0297	Ortholog(s) have serine-type endopeptidase activity
AO090005000736	-	0.0297	Ortholog(s) have mitochondrion, nucleus localization
AO090011000634	-	0.0297	Protein of unknown function

AO090120000432	-	0.0305	Ortholog of *A. nidulans* FGSC A4: AN6859, *A. fumigatus* Af293: Afu5g13070, *A. niger* CBS 513.88: An14g06090, *Aspergillus wentii*: Aspwe1_0107789 and *Aspergillus sydowii*: Aspsy1_0085559
AO090038000466	+	0.0367	Ortholog(s) have cytosol, nuclear envelope, nuclear membrane, nucleolus localization
AO090005000833	+	0.0367	Ortholog(s) have cytosol, nucleolus localization
AO090120000145	-	0.0392	Phosphoglycerate mutase; upregulated in *A. oryzae* and *A. nidulans* under hypoxic growth conditions
AO090011000656	-	0.0392	Ortholog(s) have cytoplasm localization
AO090012000887	+	0.0424	Has domain(s) with predicted FMN binding, oxidoreductase activity and role in oxidation-reduction process
AO090012000247	+	0.0451	Protein of unknown function
AO090003000415	+	0.0451	Aconitate hydratase
AO090003000750	+	0.0464	Ortholog of *A. nidulans* FGSC A4: AN2861, *A. fumigatus* Af293: Afu3g11870, *A. niger* CBS 513.88: An02g07700, *Aspergillus wentii*: Aspwe1_0105255 and *Aspergillus sydowii*: Aspsy1_0042742
AO090011000308	-	0.0496	Has domain(s) with predicted catalytic activity, glutamate-ammonia ligase activity and role in nitrogen compound metabolic process

+, Significant increase in expression in the RIB40 compared to the *ΔkojR* strain; -, Significant decrease in expression in the RIB40 compared to the *ΔkojR* strain.
Original description is based on information provided by the Comprehensive *Aspergillus oryzae* Genome Database (CAoGD) (https://nribf21.nrib.go.jp/CAoGD/ (accessed on 12 April 2023)).
