# Supplementary figures and images for: The Identification of a Target Gene of the Transcription Factor KojR and Elucidation of Its Role in Carbon Metabolism for Kojic Acid Biosynthesis in Aspergillus oryzae
Source: J Fungi (Basel). 2024 Jan 30;10(2):113. doi: 10.3390/jof10020113 (PMC10890517; doi:10.3390/jof10020113)

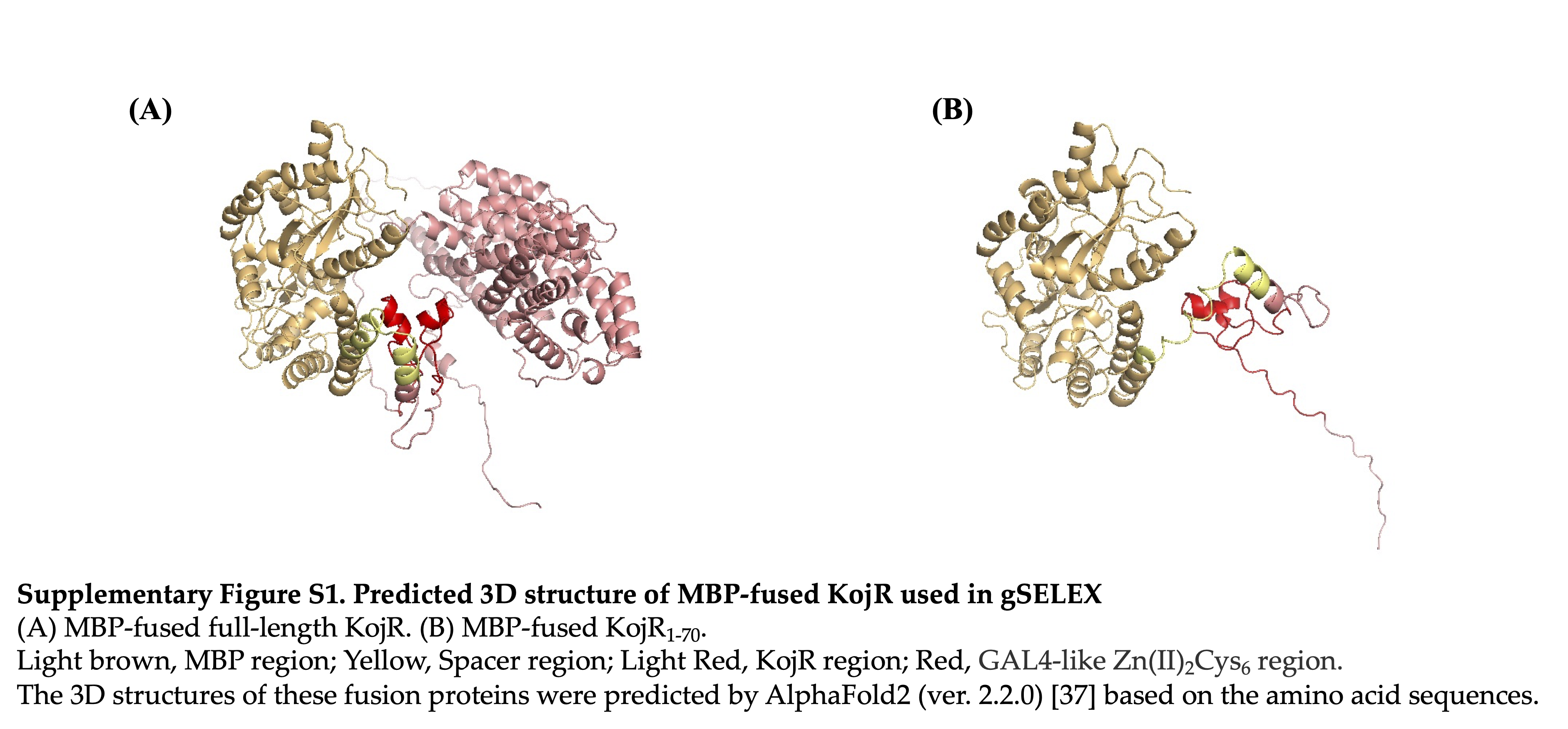

Supplement: Supplementary file 1 [file jof-10-00113-s001.zip › Supplementary Figure S1. Predicted 3D structure of MBP-fused KojR used in gSELEX..tiff]

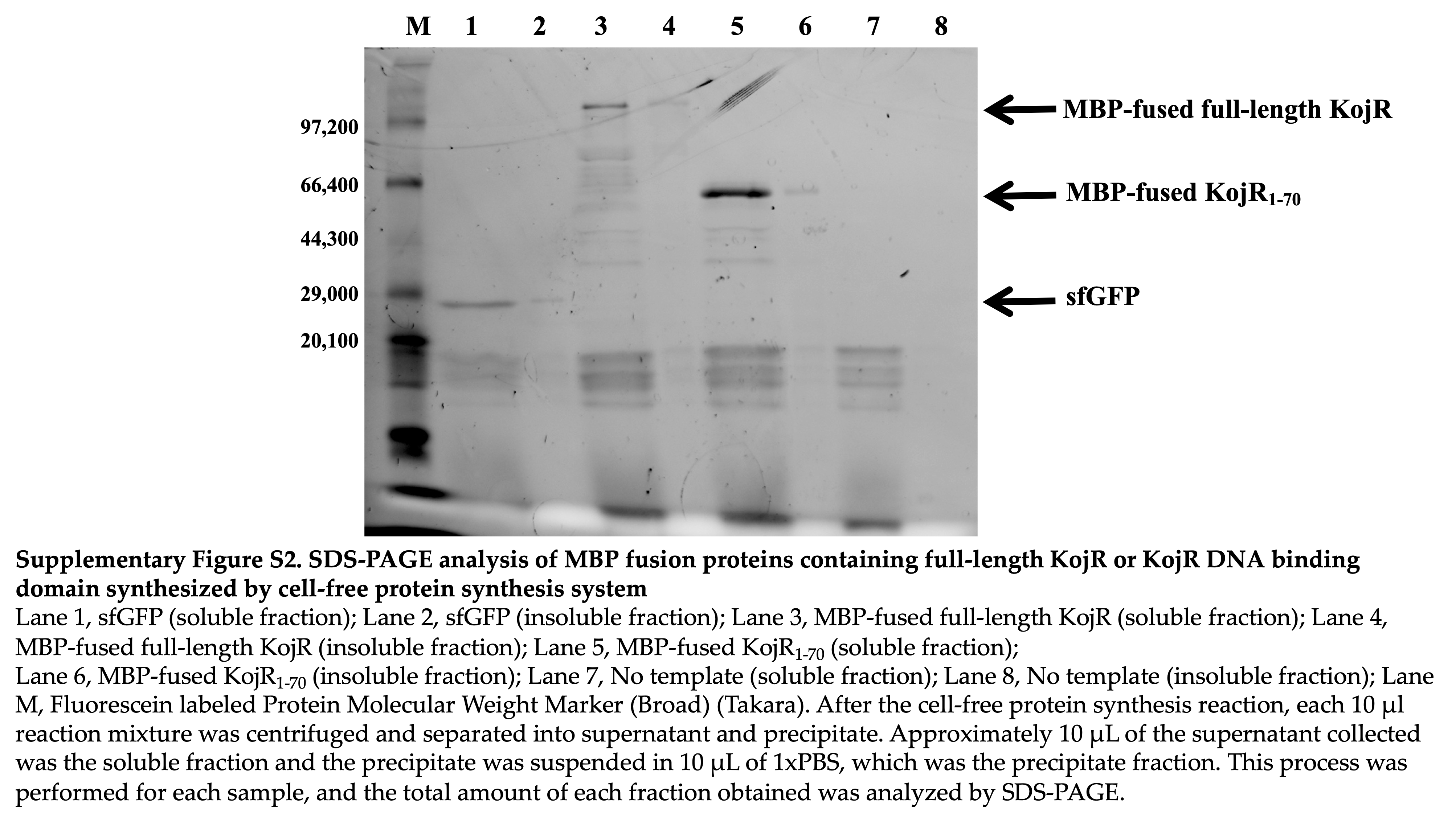

Supplement: Supplementary file 1 [file jof-10-00113-s001.zip › Supplementary Figure S2. SDS-PAGE analysis of MBP fusion proteins containing full-length KojR or KojR DNA binding domain synthesized by cell-free protein synthesis system. .tiff]

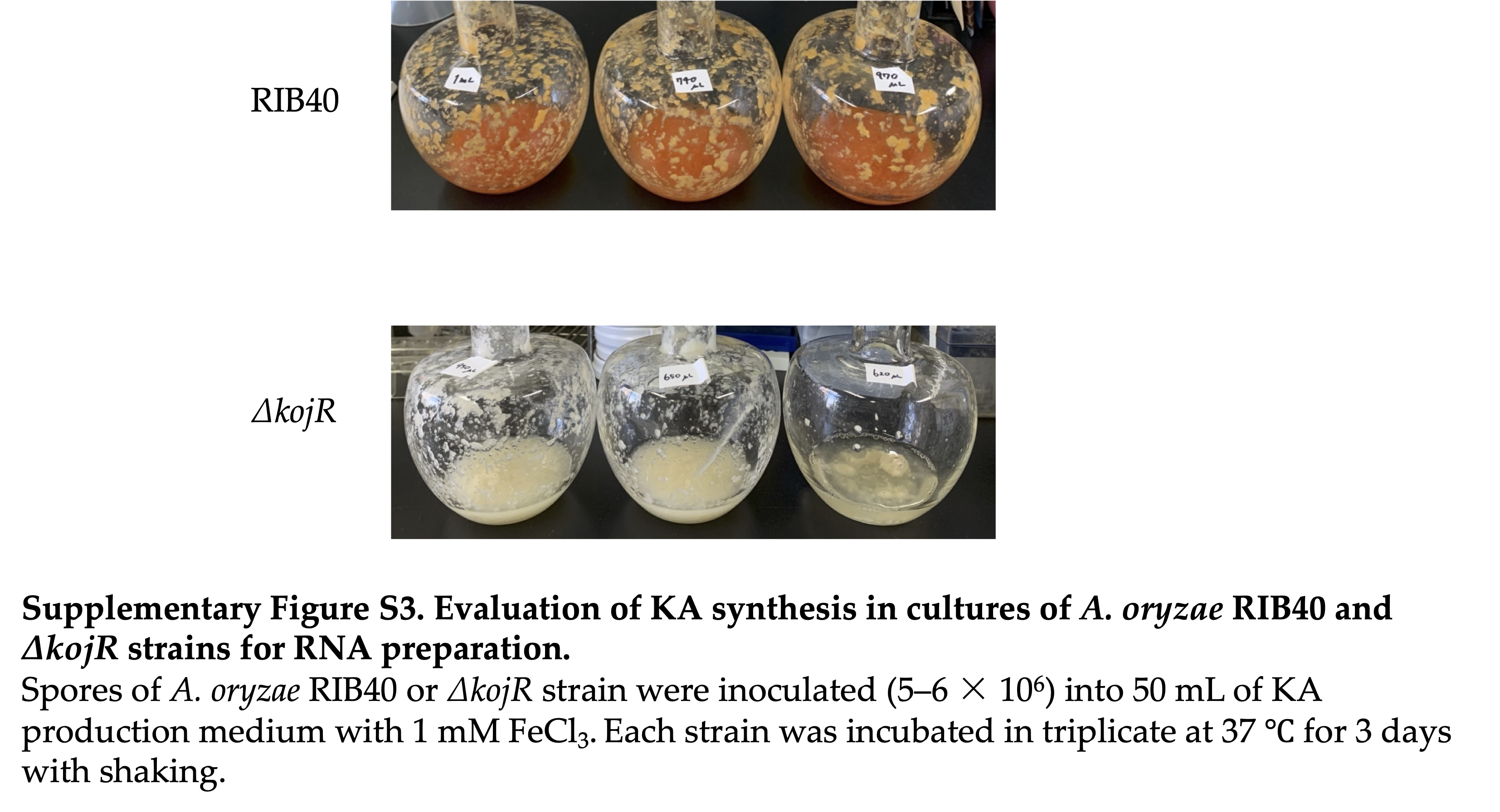

Supplement: Supplementary file 1 [file jof-10-00113-s001.zip › Supplementary Figure S3. Evaluation of KA synthesis in cultures of A. oryzae RIB40 and añkojR strains for RNA preparation..tiff]
